# Neuroendocrine Carcinoma as an Independent Prognostic Factor for Patients With Prostate Cancer: A Population-Based Study

**DOI:** 10.3389/fendo.2021.778758

**Published:** 2021-12-08

**Authors:** Jiping Yao, Yanning Liu, Xue Liang, Jiajia Shao, Yina Zhang, Jing Yang, Min Zheng

**Affiliations:** The State Key Laboratory for Diagnosis and Treatment of Infectious Diseases, Collaborative Innovation Center for Diagnosis and Treatment of Infectious Diseases, National Clinical Research Center for Infectious Diseases, The First Affiliated Hospital, College of Medicine, Zhejiang University, Hangzhou, China

**Keywords:** neuroendocrine prostate cancer, prostate adenocarcinoma, clinicopathological characteristics, prognosis, SEER, survival

## Abstract

**Background:**

Neuroendocrine carcinoma (NEC) is a rare and highly malignant variation of prostate adenocarcinoma. We aimed to investigate the prognostic value of NEC in prostate cancer.

**Methods:**

A total of 530440 patients of prostate cancer, including neuroendocrine prostate cancer (NEPC) and adenocarcinoma from 2004 to 2018 were obtained from the national Surveillance, Epidemiology, and End Results (SEER) database. Propensity score matching (PSM), multivariable Cox proportional hazard model, Kaplan‐Meier method and subgroup analysis were performed in our study.

**Results:**

NEPC patients were inclined to be older at diagnosis (Median age, 69(61-77) vs. 65(59-72), P< 0.001) and had higher rates of muscle invasive disease (30.9% vs. 9.2%, P < 0.001), lymph node metastasis (32.2% vs. 2.2%, P < 0.001), and distal metastasis (45.7% vs. 3.6%, P < 0.001) compared with prostate adenocarcinoma patients. However, the proportion of NEPC patients with PSA levels higher than 4.0 ng/mL was significantly less than adenocarcinoma patients (47.3% vs. 72.9%, P<0.001). NEPC patients had a lower rate of receiving surgery treatment (28.8% vs. 43.9%, P<0.001), but they had an obviously higher rate of receiving chemotherapy (57.9% vs. 1.0%, P<0.001). A Cox regression analysis demonstrated that the NEPC patients faced a remarkably worse OS (HR = 2.78, 95% CI = 2.34–3.31, P < 0.001) and CSS (HR = 3.07, 95% CI = 2.55–3.71, P < 0.001) compared with adenocarcinoma patients after PSM. Subgroup analyses further suggested that NEPC patients obtained significantly poorer prognosis across nearly all subgroups.

**Conclusion:**

The prognosis of NEPC was worse than that of adenocarcinoma among patients with prostate cancer. The histological subtype of NEC is an independent prognostic factor for patients with prostate cancer.

## Introduction

Prostate cancer, has the highest incidence of malignancy among men in the United States in 2021, which accounts for 26% of diagnoses (1, 2). Furthermore, it is also the second leading cause of cancer related deaths, only behind lung cancer (1). The predominant pathological type of prostate cancer is adenocarcinoma, and the assessment regarding incident rates, survival outcomes and therapeutic methods for prostate cancer are primarily according to this single histology (3). Neuroendocrine carcinoma (NEC) is a rare histological type, accounting for approximately 1% of newly diagnosed prostate cancer (4). Neuroendocrine prostate cancer (NEPC) possesses highly malignant characteristics such as poorly differentiated and high-grade (3, 5). In recent years, the incidence of NEPC has been rising and arouse wide concern (6, 7). Long-tern androgen-deprivation therapy (ADT) for prostate adenocarcinoma could contribute to castration resistant prostate cancer (CRPC), which may eventually develop to NEPC due to heterogeneity and evolution of prostate adenocarcinoma during therapy (8–11). Therefore, the extended application of ADT could partly explain the cause of the rising incidence of NEPC. Notably, the molecular mechanism by which NEPC transforms from prostate adenocarcinoma remains to be elucidated. Besides, as an increasingly recognized histologic subtype of prostate cancer, early diagnosis and effective treatment targeting specific biological characteristics for NEPC has not been developed.

Due to its rarity and a lack of associated published researches, NEPC is prone to be under-recognition and even neglected (12).However, given the upward incidence rates of NEPC in recent years as well as its refractory to medication, NEPC is attracting more attention worldwide increasingly. Currently, studies about NEPC were mainly case reports or retrospective researches based on small sample data. Therefore, our study compared NEPC with prostate adenocarcinoma comprehensively based on large population, aiming to overcome the remarkable challenges in the clinical treatment of patients with the rare subtype of prostate cancer. We utilized the national Surveillance, Epidemiology, and End Results (SEER) database (2004–2018) to compare the clinicopathological characteristics and survival outcomes between NEPC and prostate adenocarcinoma, the most common histological type of prostate cancer. Furthermore, we investigated the prognostic value of NEPC for patients with prostate cancer.

## Materials and Methods

### Patients

This retrospective cohort study was conducted *via* the SEER database of the National Cancer Institute (http://seer.cancer.gov/). A total of 530440 patients of prostate cancer, including NEC and adenocarcinoma from 2004 to 2018 were obtained from the latest version of the SEER 18 database, as released in November 2020, using the SEER*Stat software (version 8.3.9). We identified prostate cancer according to the International Classification of Diseases for Oncology (Third Edition, ICD‐O‐3). NEPC, a generalized NEC of prostate, are classified by the American Joint Committee on Cancer (AJCC) as four histological subtypes, mainly including large cell neuroendocrine carcinoma (LCNE, ICD-0-3 codes 8013/3), small cell carcinoma (SCC, ICD-0-3 codes 8041/3), neuroendocrine carcinoma not otherwise specified (NEC NOS, ICD-0-3 codes 8246/3), and neuroendocrine differentiation (NED, ICD-0-3 codes 8574/3). And adenocarcinoma (ICD-0-3 code 8140/3) were included for comparison. All patients included were diagnosed by positive histology. Meanwhile, the exclusion criteria of patients were: (1) the information of age, race, marital status, survival time, surgery, radiotherapy, chemotherapy is unknown; (2) not the first tumor; (3) survival time < 1 month; (4) age at diagnosis < 18 years old; (5) with multiple primary tumor sites; (6) autopsy or death certificate only.

### Clinical Variables

Variables covered demographic information (e.g., race, age at diagnosis, marital status and year of diagnosis), tumor characteristics [e.g., grade, tumor-node-metastasis (TNM) stage, lymph nodes and prostate‐specific antigen (PSA)], treatment (e.g., surgery, radiation and chemotherapy), and survival information (survival months and vital status). In the SEER database, age is code as 18-59 years old, 60-74 years old and ≥75 years old. Race is coded as white, black, or other (e.g., American Indian/Alaskan native or Asian/Pacific Islander). Marital status is coded as married and not married. Between 2004 and 2018, patients were categorized according to 6^th^ editions of the TNM classification. PSA was divided into four levels including 0-4.0 ng/ml, 4.1-10.0 ng/ml, 10.1-20 ng/ml, >20 ng/ml and unknown. We also enrolled treatment modality including surgery, chemotherapy, and radiation therapy information, which were divided with “Yes” and “No”. The main outcome in this study were overall survival (OS) and cancer specific survival (CSS) according to data in the SEER database. OS was defined as the time interval from diagnosis to death for any cause or last follow-up. CSS refer to death from NEPC or prostate adenocarcinoma based on the recorded cause of death.

### Statistical Analysis

Baseline demographic and clinicopathologic characteristics were performed to assess whether the distribution of the study population had significant differences between NEPC and prostate adenocarcinoma. Pearson’s chi-square tests were adopted to calculate the differences in the distribution. We used Kaplan‐Meier method and log‐rank test to compare OS and CSS among patients with the two histological subtypes of prostate cancer. In order to overcome the effect of patient confounding bias, propensity score matching (PSM) method was adopted to remove the potential impact. Covariates of the two histological subtypes groups were matched with a ratio of 1:1 (R package “MatchIt”). The multivariable Cox proportional hazard model was performed to calculate hazard ratios (HR) and 95% confidence intervals (95% CI) according to histological types. We established two adjusted models in Cox regression analysis, in which covariates including age at diagnosis, marital status, lymph nodes examined, lymph nodes positive, PSA and TNM stage were adjusted. We stratified the two histological subtypes groups based on the covariates into subgroups and applied stratified analyses to determine the subgroups that contribute to survival disadvantage of NEC. Interaction between the subgroups was calculated by R studio. The forest plot was applied to compare the impact of NEC and adenocarcinoma to survival outcomes of prostate cancer patients. Multivariate regression analysis was used to conduct subgroup analyses. Statistical analyses were performed using IBM SPSS Statistics 23.0 (IBM Corp., Armonk, NY, USA) and R version 4.0.3 (R Foundation for Statistical Computing, Vienna, Austria). Two-sided P values < 0.05 were considered as the threshold to define statistical significance.

## Result

### Patient Characteristics

**Figure 1:**
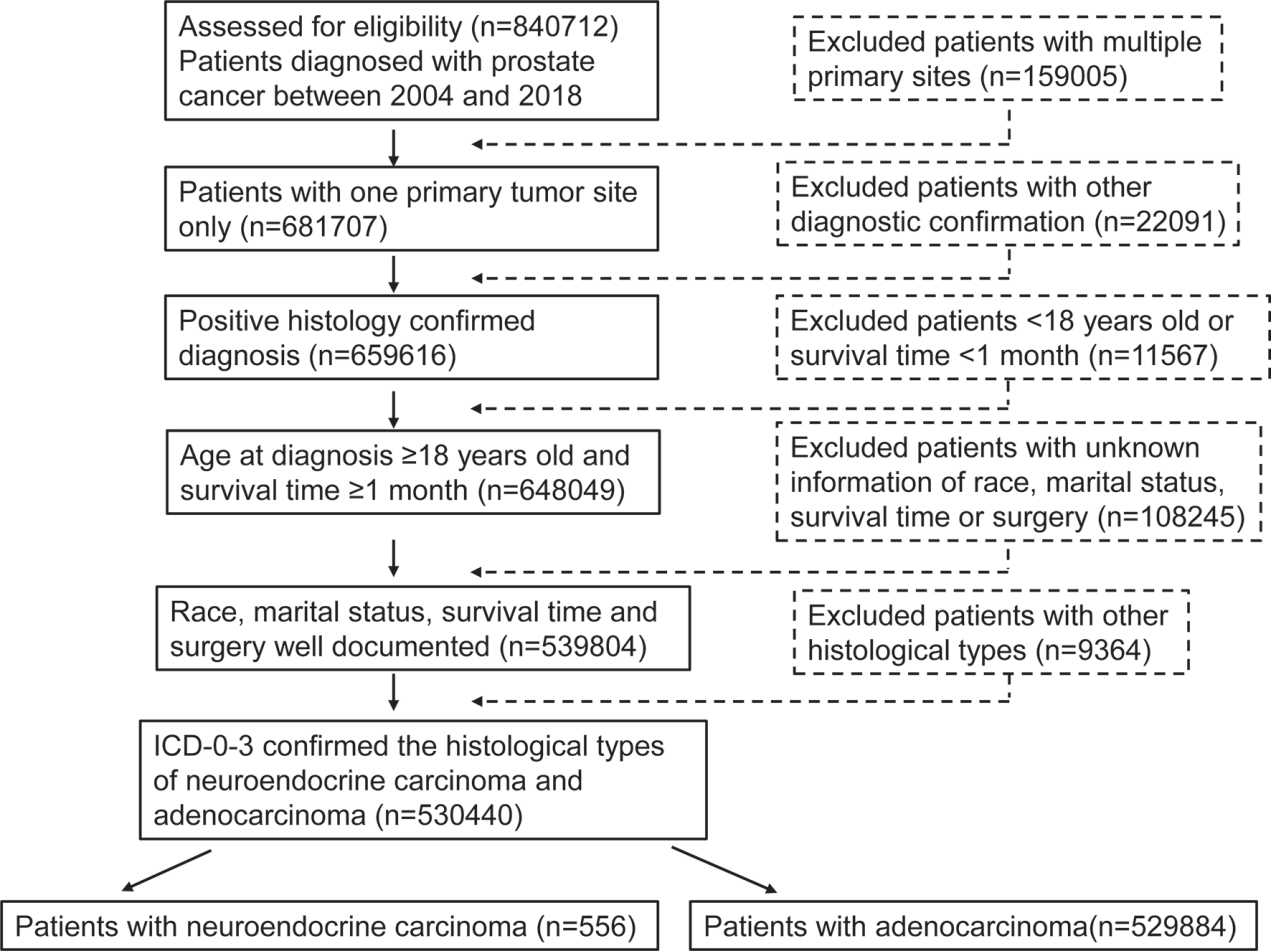
Flowchart of patient selection steps.

This study enrolled 530440 eligible prostate cancer patients including 556 patients with NEPC and 529884 patients with prostate adenocarcinoma from SEER database between 2004 and 2018 (**Figure 1**). **Table 1** summarize the baseline demographic and clinicopathologic characteristics of these patients. The age at diagnosis of NEC patients were inclined to be older compared with adenocarcinoma patients, median age at diagnosis 69(61-77) vs. 65(59-72), age≥75 years (29.5% vs. 16.3%). The incidence of NEC in patients newly diagnosed were increasing roughly during our study period whereas the incidence of adenocarcinoma remained stable. Significantly, the NEC patients formed a higher proportion with a more advanced stage than the adenocarcinoma patients (59.6% vs. 12.2%, P<0.001), as displayed by a higher proportion of muscle invasive disease (30.9% vs. 9.2%, P<0.001), lymph node metastasis (32.2% vs. 2.2%, P<0.001), and distal metastasis (45.7% vs. 3.6%, P < 0.001). Lymph nodes were more likely to be examined in adenocarcinoma patients (11.2% vs. 24.7%, P<0.001) whereas positive lymph nodes were more common in the NEC patients (9.2% vs. 1.6%, P <0.001). Additionally, NEC patients with PSA levels higher than 4.0 ng/mL accounted for 47.3%, compared with 72.9% of adenocarcinoma patients. Furthermore, NEC patients had a lower rate of receiving surgery treatment compared with adenocarcinoma patients (28.8% vs. 43.9%, P<0.001). However, NEC patients were prone to receiving chemotherapy treatment, which accounted for 57.9% compared with 1.0% of adenocarcinoma patients. There was no significant difference of radiation between NEC and adenocarcinoma patients.

**Table 1:** Baseline demographic and clinicopathologic characteristics of patients with prostate adenocarcinoma compare to NEPC.

Characteristics	NEPC (n = 556)	Prostate Adenocarcinoma (n = 529884)	P value
**Median age, y (IQR)**	69(61-77)	65(59-72)	<0.001
**Age at diagnosis, n (%)**			<0.001
18-59	115(20.7)	138473(26.1)	
60-74	281(50.5)	304879(57.5)	
≥75	160(28.8)	86532(16.3)	
**Race, n (%)**			0.001
White	464(83.5)	414093(78.1)	
Black	57(10.3)	86273(16.3)	
Other	35(6.2)	29518(5.6)	
**Marital status, n (%)**			<0.001
Married	377(67.8)	395936(74.7)	
Not married	179(32.2)	133948(25.3)	
**Year of diagnosis, n (%)**			<0.001
2004-2008	117(21.0)	181794(34.3)	
2009-2013	203(36.5)	178887(33.8)	
2014-2018	236(42.5)	169203(31.9)	
**Grade, n (%)**			<0.001
G1	1(0.2)	40933(7.7)	
G2	12(2.2)	213722(40.3)	
G3	240(43.2)	226212(42.7)	
G4	41(7.4)	876(0.2)	
Unknown	262(47.1)	48141(9.1)	
**Stage, n (%)**			<0.001
I	0(0.0)	1478(0.3)	
II	45(8.1)	340943(64.3)	
III	7(1.3)	35235(6.6)	
IV	324(58.3)	29774(5.6)	
Unknown	180(32.4)	122454(23.1)	
**T stage, n (%)**			<0.001
T1	52(9.4)	161210(30.4)	
T2	92(16.5)	203640(38.4)	
T3	50(9.0)	41566(7.8)	
T4	122(21.9)	7284(1.4)	
Unknown	207(43.2)	116184(21.9)	
**N stage, n (%)**			<0.001
N0	152(27.3)	393696(74.3)	
N1	179(32.2)	11647(2.2)	
Unknown	225(40.5)	124541(23.5)	
**M stage, n (%)**			<0.001
M0	122(21.9)	394657(74.5)	
M1	254(45.7)	18815(3.6)	
Unknown	180(32.4)	116412(22.0)	
**Lymph nodes examined, n (%)**			<0.001
None	471(84.7)	393040(74.2)	
More than one	62(11.2)	130720(24.7)	
Unknown	23(4.1)	6124(1.2)	
**Lymph nodes positive, n (%)**			<0.001
None	16(2.9)	122138(23.0)	
More than one	51(9.2)	8448(1.6)	
Unknown	489(87.9)	399298(75.4)	
**PSA, ng/mL, n (%)**			<0.001
0‐4.0	127(22.8)	57511(10.9)	
4.1-10.0	92(16.5)	265323(50.1)	
10.1-20.0	42(7.6)	66929(12.6)	
>20.0	129(23.2)	53942(10.2)	
Unknown	166(29.9)	86179(16.3)	
**Surgery**			<0.001
No	396(71.2)	297267(56.1)	
Cryoprostatectomy	0(0)	4875(0.9)	
Laser ablation	3(0.5)	1202(0.2)	
TURP	116(20.9)	25297(4.8)	
Partial prostatectomy	2(0.4)	1123(0.2)	
Radical prostatectomy	39(7.0)	200120(37.8)	
**Radiation**			<0.001
No	349(62.8)	343593(64.8)	
Beam radiation	199(35.8)	128148(24.2)	
Radioactive implants	3(0.5)	34974(6.6)	
Combination of beam with implants or isotopes	1(0.2)	20895(3.9)	
Radioisotopes	0(0)	871(0.2)	
Radiation method unknown	4(0.7)	1404(0.3)	
**Chemotherapy**			<0.001
No	234(42.1)	524571(99.0)	
Yes	322(57.9)	5313(1.0)	
**Overall mortality**			<0.001
Alive	113(20.3)	431549(81.4)	
Dead	443(79.7)	98335(18.6)	
**Cause special mortality**			<0.001
Alive	150(27.0)	495892(93.6)	
Dead	406(73.0)	33992(6.4)	

PSA, prostate‐specific antigen; NEPC, neuroendocrine prostate cancer; IQR, interquartile range; TURP, Transurethral resection of prostate.

NEPC are defined by AJCC as different histological subtypes, including LCNE, SCC, NEC NOS, and NED. The first three are *de novo* NEPC while NED originated from the trans-differentiation of adenocarcinoma during the process of resistance to ATD or androgen receptor pathway inhibitors (ARPIs) treatment. The results of comparison among the four histological subtypes of NEPC and prostate adenocarcinoma are summarized in **Table 2**. The four histological subtypes patients all had higher proportions of muscle invasive disease (LCNE 50.0% vs. SCC 30.7% vs. NEC NOS 31.0% vs. NED 30.4%), lymph node metastasis (LCNE 50.0% vs. SCC 31.4% vs. NEC NOS 34.1% vs. NED 31.2%), and distal metastasis (LCNE 66.7% vs. SCC 46.6% vs. NEC NOS 45.7% vs. NED 42.4%), as compared to prostate adenocarcinoma patients (9.2%, 2.2%, 3.6%) respectively. Three histological subtypes of NEPC patients had low rates to receiving surgery treatment (SCC 25.0% vs. NEC NOS 31.0% vs. NED 32.8%) than that of adenocarcinoma (43.9%) except for LCNE (83.3%). However, the proportions of receiving radiation treatment of SCC (38.5%), NEC NOS (36.4%), and NED 33.6%) had no significant difference as compared to adenocarcinoma (35.2%) except for LCNE (66.7%). Additionally, the proportions of receiving chemotherapy treatment of LCNE (50.0%), SCC (67.9%) and NEC NOS (55.0%) patients were obviously higher than that of adenocarcinoma patients (1.0%) while NED patients (37.6%) were between *de novo* NEPC and prostate adenocarcinoma patients. Notably, NED patients with PSA levels higher than 4.0 ng/mL accounted for 72.0%, which was significantly higher than that of the other three histological subtypes of NEPC patients (LCNE 50.0%, SCC 36.9%, NEC NOS 47.3%). We speculated that it may attributed to the mixed adenocarcinoma and NEC components of NED.

**Table 2:** Baseline demographic and clinicopathologic characteristics of patients with prostate adenocarcinoma compare to four histological subtypes of NEPC.

Characteristics	Prostate adenocarcinoma (n = 529884)	NEPC (n = 556)				P value
		LCNE (n = 6)	SCC (n = 296)	NEC NOS (n = 129)	NED (n = 125)	
**Age at diagnosis, y, n (%)**						<0.001
18-59	138473(26.1)	3(50.0)	56(18.9)	32(24.8)	24(19.2)	
60-74	304879(57.5)	1(16.7)	151(51.0)	58(45.0)	71(56.8)	
≥75	86532(16.3)	2(33.3)	89(30.1)	39(30.2)	30(24.0)	
**Race, n (%)**						0.020
White	414093(78.1)	5(83.3)	244(82.4)	107(82.9)	108(86.4)	
Black	86273(16.3)	0(0)	32(10.8)	13(10.1)	12(9.6)	
Other	29518(5.6)	1916.7)	20(6.8)	9(7.0)	5(4.0)	
**Marital status, n (%)**						0.002
Married	395936(74.7)	3(50.0)	208(70.3)	86(66.7)	80(64.0)	
Not married	133948(25.3)	3(50.0)	88(29.7)	43(33.3)	45(36.0)	
**Year of diagnosis, n (%)**						<0.001
2004-2008	181794(34.3)	1(16.7)	57(19.3)	37(28.7)	22(17.6)	
2009-2013	178887(33.8)	3(50.0)	109(36.8)	48(37.2)	43(34.4)	
2014-2018	169203(31.9)	2(33.3)	130(43.9)	44(34.1)	60(48.0)	
**Grade, n (%)**						<0.001
G1	40933(7.7)	0(0)	0(0)	0(0)	1(0.8)	
G2	213722(40.3)	0(0)	6(2.0)	2(1.6)	4(3.2)	
G3	226212(42.7)	1(16.7)	79(26.7)	72(55.8)	88(70.4)	
G4	876(0.2)	1(16.7)	27(9.1)	11(8.5)	2(1.6)	
Unknown	48141(9.1)	4(66.7)	184(62.2)	44(34.1)	30(24.0)	
**Stage, n (%)**						<0.001
I	1478(0.3)	0(0)	0(0)	0(0)	0(0)	
II	340943(64.3)	0(0)	22(7.4)	13(10.1)	10(8.0)	
III	35235(6.6)	0(0)	1(0.3)	2(1.6)	4(3.2)	
IV	29774(5.6)	5(83.3)	176(59.5)	74(57.4)	69(55.2)	
Unknown	122454(23.1)	1(16.7)	97(32.8)	40(31.0)	42(33.6)	
**T stage, n (%)**						<0.001
T1	161210(30.4)	0(0)	27(9.1)	13(10.1)	12(9.6)	
T2	203640(38.4)	1(16.7)	55(18.6)	19(14.7)	17(13.6)	
T3	41566(7.8)	0(0)	27(9.1)	9(7.0)	14(11.2)	
T4	7284(1.4)	3(50.0)	64(21.6)	31(24.0)	24(19.2)	
Unknown	116184(21.9)	2(33.3)	123(41.6)	57(44.2)	58(46.4)	
**N stage, n (%)**						<0.001
N0	393696(74.3)	0(0)	84(28.4)	34(26.4)	34(27.2)	
N1	11647(2.2)	3(50.0)	93(31.4)	44(34.1)	39(31.2)	
Unknown	124541(23.5)	3(50.0)	119(40.2)	51(39.5)	52(41.6)	
**M stage, n (%)**						<0.001
M0	394657(74.5)	1(16.7)	60(20.3)	32(24.8)	29(23.2)	
M1	18815(3.6)	4(66.7)	138(46.6)	59(45.7)	53(42.4)	
Unknown	116412(22.0)	1(16.7)	98(33.1)	38(29.5)	43(34.4)	
**Lymph nodes examined, n (%)**						<0.001
None	393040(74.2)	6(100.0)	258(87.2)	102(79.1)	105(84.0)	
More than one	130720(24.7)	0(0)	25(8.4)	19(14.7)	18(14.4)	
Unknown	6124(1.2)	0(0)	13(4.4)	8(6.2)	2(1.6)	
**Lymph nodes positive, n (%)**						<0.001
None	122138(23.0)	0(0)	7(2.4)	3(2.3)	6(4.8)	
More than one	8448(1.6)	0(0)	21(7.1)	16(12.4)	14(11.2)	
Unknown	399298(75.4)	6(100.0)	268(90.5)	110(85.3)	105(84.0)	
**PSA, ng/mL, n (%)**						<0.001
0‐4.0	57511(10.9)	1(16.7)	81(27.4)	32(24.8)	13(10.4)	
4.1-10.0	265323(50.1)	1(16.7)	47(15.9)	17(13.2)	27(21.6)	
10.1-20.0	66929(12.6)	0(0)	18(6.1)	13(10.1)	11(8.8)	
>20.0	53942(10.2)	2(33.3)	44(14.9)	31(24.0)	52(41.6)	
Unknown	86179(16.3)	2(33.3)	106(35.8)	36(27.9)	22(17.6)	
**Surgery**						<0.001
No	297267(56.1)	1(16.7)	222(75.0)	89(69.0)	84(67.2)	
Cryoprostatectomy	4875(0.9)	0(0)	0(0)	0(0)	0(0)	
Laser ablation	1202(0.2)	0(0)	1(0.3)	0(0)	2(1.6)	
TURP	25297(4.8)	5(83.3)	58(19.6)	29(22.5)	24(19.2)	
Partial prostatectomy	1123(0.2)	0(0)	2(0.7)	0(0)	0(0)	
Radical prostatectomy	200120(37.8)	0(0)	13(4.4)	11(8.5)	15(12.0)	
**Radiation**						<0.001
No	343592(64.8)	2(33.3)	182(61.5)	82(63.6)	83(66.4)	
Beam radiation	128148(24.2)	4(66.7)	109(36.8)	46(35.7)	40(32.0)	
Radioactive implants	34974(6.6)	0(0)	3(1.0)	0(0)	0(0)	
Combination of beam with implants or isotopes	20895(3.9)	0(0)	0(0)	0(0)	1(0.8)	
Radioisotopes	871(0.2)	0(0)	0(0)	0(0)	0(0)	
Radiation method unknown	1404(0.3)	0(0)	2(0.7)	1(0.8)	1(0.8)	
**Chemotherapy**						<0.001
No	524571(99.0)	3(50.0)	95(32.1)	58(45.0)	78(62.4)	
Yes	5313(1.0)	3(50.0)	201(67.9)	71(55.0)	47(37.6)	
**Overall mortality**						<0.001
Alive	431549(81.4)	1(16.7)	48(16.2)	21(16.3)	43(34.4)	
Dead	98335(18.6)	5(83.3)	248(83.8)	108(83.7)	82(65.6)	
**Cause special mortality**						<0.001
Alive	495892(93.6)	1(16.7)	65(22.0)	31(24.0)	53(42.4)	
Dead	33992(6.4)	5(83.3)	231(78.0)	98(76.0)	72(57.6)	

PSA, prostate‐specific antigen; NEPC, neuroendocrine prostate cancer; TURP, Transurethral resection of prostate; LCNE, large cell neuroendocrine carcinoma; SCC, small cell carcinoma; NEC NOS, neuroendocrine carcinoma not otherwise specified; NED, neuroendocrine differentiation.

### Survival Analyses

**Figure 2:**
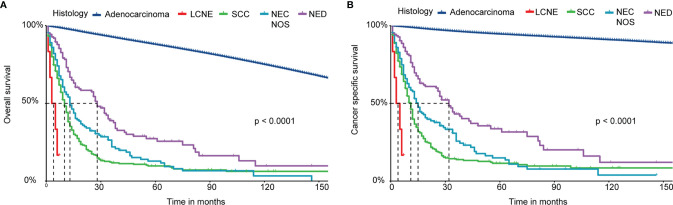
Survival analysis of OS and CSS for patients with four histological subtypes of NEPC and prostate adenocarcinoma. **(A)** Kaplan-Meier analysis of OS; **(B)** Kaplan-Meier analysis of CSS. NEPC, neuroendocrine prostate cancer; LCNE, large cell neuroendocrine carcinoma; SCC, small cell carcinoma; NEC NOS, neuroendocrine carcinoma not otherwise specified; NED, neuroendocrine differentiation; OS, overall survival; CSS, cancer-specific survival.

We performed Kaplan‐Meier curves to compare the OS and CSS between the four histological subtypes of NEC and adenocarcinoma patients (**Figure 2**). The LCNE patients had the worst OS and CSS among all histological subtypes, followed by SCC, NEC NOS, NED, and adenocarcinoma patients. Intriguingly, these results suggested that the OS and CSS of NED patients were better than that of *de novo* NEC patients but worse than that of adenocarcinoma patients. Furthermore, we performed the survival analysis of 1-, 2-, 3-, 4- and 5-year OS and CSS rates of patents with the four histological subtypes of NEPC and prostate adenocarcinoma (**Table 3**). The LCNE patients had the worst 5-year OS rate among all histological subtypes, followed by SCC, NEC NOS, NED, and adenocarcinoma patients. Compared with the four histological subtypes of NEPC, the 1-, 3- and 5-year OS rate of adenocarcinoma (97.7%, 92.7%, 88.0%) roughly remained stable. The 1-, 2-, 3-, 4- and 5-year CSS revealed the similar outcomes.

**Table 3:** Overall survival and cancer specific survival of patients with NEPC and prostate adenocarcinoma.

	Overall survival					Cancer specific survival				
	Year	Prostate					NEPC				Prostate					NEPC			
		Adenocarcinoma	LCNE	SCC	NEC NOS	NED	Adenocarcinoma	LCNE	SCC	NEC NOS	NED
1	97.7 (97.7-97.8)	0	38.1 (32.8-44.3)	54.0 (46.0-63.5)	70.4 (62.6-79.2)	98.8 (98.8-98.9)	0	39.9 (34.4-46.2)	57.6 (49.6-67.0)	73.5 (65.8-82.0)
2	95.2 (95.1-95.2)	0	19.1 (14.9-24.5)	32.3 (24.9-41.9)	57.4 (48.9-67.3)	97.5 (97.5-97.6)	0	20.5 (16.0-26.2)	37.7 (29.8-47.9)	59.9 (51.4-69.8)
3	92.7 (92.7-92.8)	0	11.7 (8.3-16.5)	22.0 (15.4-31.4)	38.9 (30.4-49.7)	96.4 (96.4-96.5)	0	13.8 (10.0-19.1)	25.7 (18.3-36.1)	43.2 (34.2-54.5)
4	90.3 (90.2-90.4)	0	10.8 (7.5-15.5)	15.1 (9.5-24.1)	28.8 (20.9-39.8)	95.5 (95.4-95.6)	0	12.7 (9.0-17.9)	17.7 (11.2-27.8)	35.6 (26.7-47.4)
5	88.0 (87.9-88.1)	0	9.7 (6.6-14.4)	11.6 (6.7-20.2)	25.6 (17.8-36.8)	94.7 (94.6-94.8)	0	11.5 (7.9-16.6)	13.6 (7.9-23.4)	31.6 (22.7-44.0)

NEPC, neuroendocrine prostate cancer; LCNE, large cell neuroendocrine carcinoma; SCC, small cell carcinoma; NEC NOS, neuroendocrine carcinoma not otherwise specified; NED, neuroendocrine differentiation.

Due to the imbalanced basic demographic and clinicopathologic characteristics, we conducted PSM *via* R software to minimize confounding factors. All the covariates in the present study were matched between the two groups. The baseline after PSM was shown in **Table 4**. We matched 401 NEPC patients with 401 prostate adenocarcinoma patients with a ratio of 1:1. After eliminating the selection bias, all variables were matched as defined by the P value >0.05. We performed multivariable Cox proportional hazard regression based on a non-adjusted model and three adjusted models (**Table 5**). Adjusted I model adjusts for age, marital status, lymph nodes examined and lymph nodes positive and adjusted II model adjusted for age, marital status, lymph nodes examined and lymph nodes positive, T stage, N stage, M stage, PSA level. NEPC patients faced a remarkably worse OS (HR = 2.78, 95% CI = 2.34–3.31, P < 0.001) and CSS (HR = 3.07, 95% CI = 2.55–3.71, P < 0.001) compared with prostate adenocarcinoma patients. These findings emphasized the worse survival outcomes for the histological subtype of NEC.

**Table 4:** Propensity score matching for baseline factors.

Characteristics	Prostate Adenocarcinoma (n = 484)	NEPC (n = 484)	P value
**Age at diagnosis, y, n (%)**			0.722
18-59	92 (19.0)	101 (20.9)	
60-74	249 (51.4)	248 (51.2)	
≥75	143 (29.5)	135 (27.9)	
**Race, n (%)**			0.222
White	382 (78.9)	396 (81.8)	
Black	74 (15.3)	56 (11.6)	
Other	28 (5.8)	32 (6.6)	
**Marital status, n (%)**			0.500
Married	310 (64.0)	321 (66.3)	
Not married	174 (36.0)	163 (33.7)	
**Year of diagnosis, n (%)**			0.006
2004-2006	127 (26.2)	97 (20.0)	
2007-2009	137 (28.3)	180 (37.2)	
2010-2012	220 (45.5)	207 (42.8)	
**Grade, n (%)**			0.004
G1	2 (0.4)	1 (0.2)	
G2	23 (4.8)	12 (2.5)	
G3	234 (48.3)	237 (49.0)	
G4	11 (2.3)	33 (6.8)	
Unknown	214 (44.2)	201 (41.5)	
**Stage, n (%)**			NaN
I	0 (0.0)	0 (0.0)	
II	50 (10.3)	45 (9.3)	
III	20 (4.1)	7 (1.4)	
IV	210 (43.4)	270 (55.8)	
Unknown	204 (42.1)	162 (33.5)	
**T stage, n (%)**			0.011
T1	56 (11.6)	48 (9.9)	
T2	83 (17.1)	76 (15.7)	
T3	50 (10.3)	42 (8.7)	
T4	58 (12.0)	99 (20.5)	
Unknown	237 (49.0)	219 (45.2)	
**N stage, n (%)**			<0.001
N0	172 (35.5)	127 (26.2)	
N1	88 (18.2)	149 (30.8)	
Unknown	224 (43.9)	208 (49.1)	
**M stage, n (%)**			0.006
M0	137 (28.3)	106 (21.9)	
M1	168 (34.7)	214 (44.2)	
Unknown	179 (37.0)	164 (33.9)	
**Lymph nodes examined, n (%)**			0.245
None	405 (83.5)	405 (83.5)	
More than one	47 (9.7)	57 (11.8)	
Unknown	32 (6.6)	22 (4.5)	
**Lymph nodes positive, n (%)**			0.188
None	19 (3.9)	16 (3.3)	
More than one	31 (6.4)	46 (9.5)	
Unknown	434 (89.7)	422 (87.2)	
**PSA, ng/mL, n (%)**			0.012
0‐4.0	65 (13.4)	83 (17.1)	
4.1-10.0	90 (18.6)	76 (15.7)	
10.1-20.0	43 (8.9)	37 (7.6)	
>20.0	160(33.1)	125 (25.8)	
Unknown	126 (26.0)	163 (33.7)	
**Surgery**			0.003
No	367 (75.8)	339 (70.0)	
Cryoprostatectomy	2 (0.4)	0 (0)	
Laser ablation	0(0)	2(0.4)	
TURP	64(13.2)	106(21.9)	
Partial prostatectomy	2(0.4)	1(0.2)	
Radical prostatectomy	49(10.1)	36(7.4)	
**Radiation**			0.046
No	327 (67.6)	310 (64.0)	
Beam radiation	139 (28.7)	166 (34.3)	
Radioactive implants	8(1.7)	3(0.6)	
Combination of beam with implants or isotopes	7(1.4)	1(0.2)	
Radioisotopes	1(0.2)	0(0)	
Radiation method unknown	2(0.4)	4(0.8)	
**Chemotherapy**			0.479
No	246 (50.8)	234 (48.3)	
Yes	238 (49.2)	250 (51.7)	
**Overall mortality**			<0.001
Alive	241 (49.8)	105 (21.7)	
Dead	243 (50.2)	379 (78.3)	
**Cause special mortality**			<0.001
Alive	294 (60.7)	141 (29.1)	
Dead	190 (39.3)	343 (70.9)	

PSA, prostate‐specific antigen; TURP, Transurethral resection of prostate; NEPC, neuroendocrine prostate cancer.

**Table 5:** Multivariable Cox proportional hazard model.

Outcomes	NEPC HR (95% CI)	P-value
Overall survival		
Non-adjusted	23.20 (21.02-25.60)	<0.001
Adjust I	19.24 (17.43-21.23)	<0.001
Adjust II	6.35 (5.75-7.02)	<0.001
PSM	2.78 (2.34-3.31)	<0.001
Cancer specific survival		
Non-adjusted	48.08 (43.38-52.29)	<0.001
Adjust I	34.65 (31.24-38.43)	<0.001
Adjust II	7.70 (6.94-8.56)	<0.001
PSM	3.07 (2.55-3.71)	<0.001

PSM, propensity score matching; NEPC, neuroendocrine prostate cancer. HR, hazard ratios.

### Subgroup Analyses

**Figure 3:**
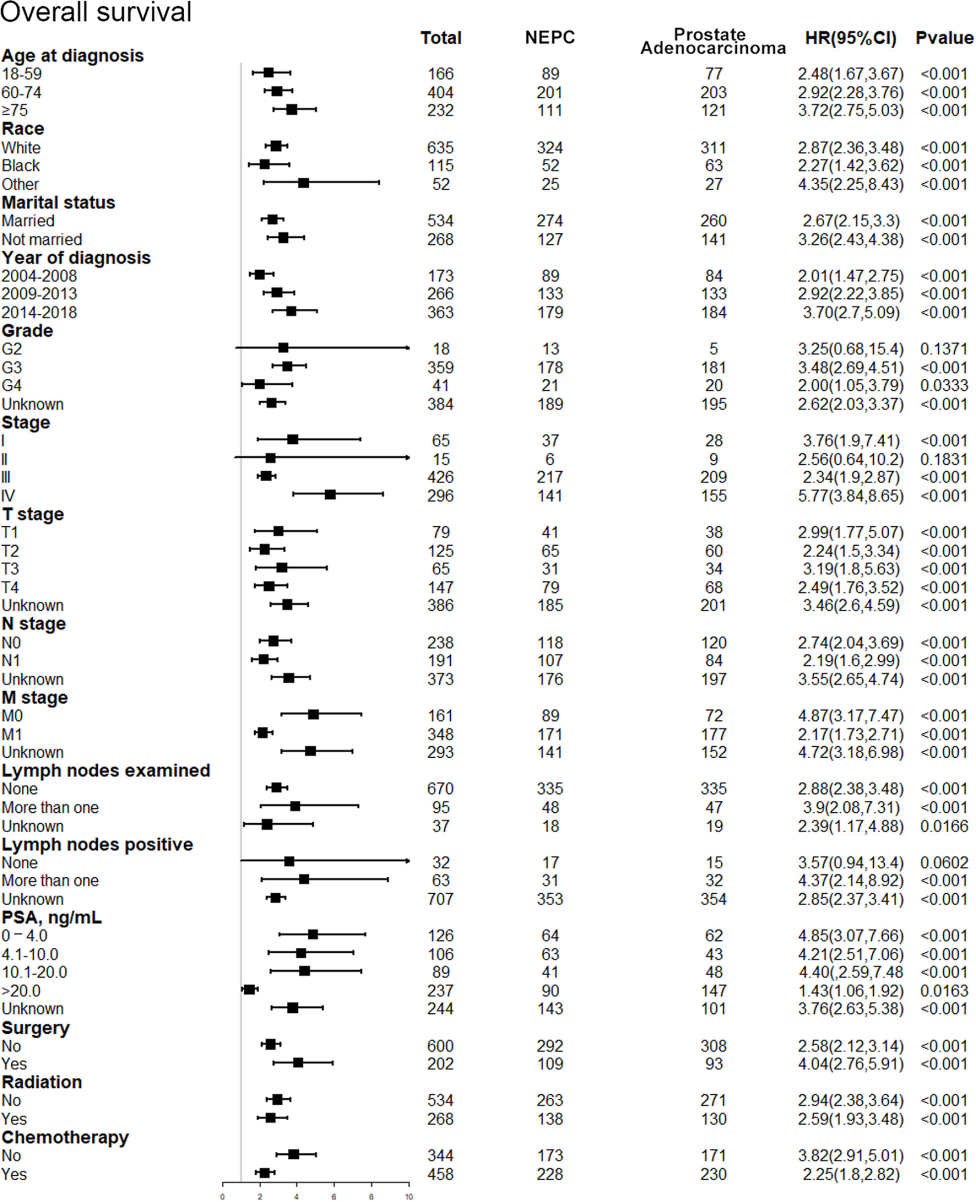
Forest plot of the subgroup analysis for NEPC and prostate adenocarcinoma in OS. NEPC, neuroendocrine prostate cancer; OS, overall survival.

**Figure 4:**
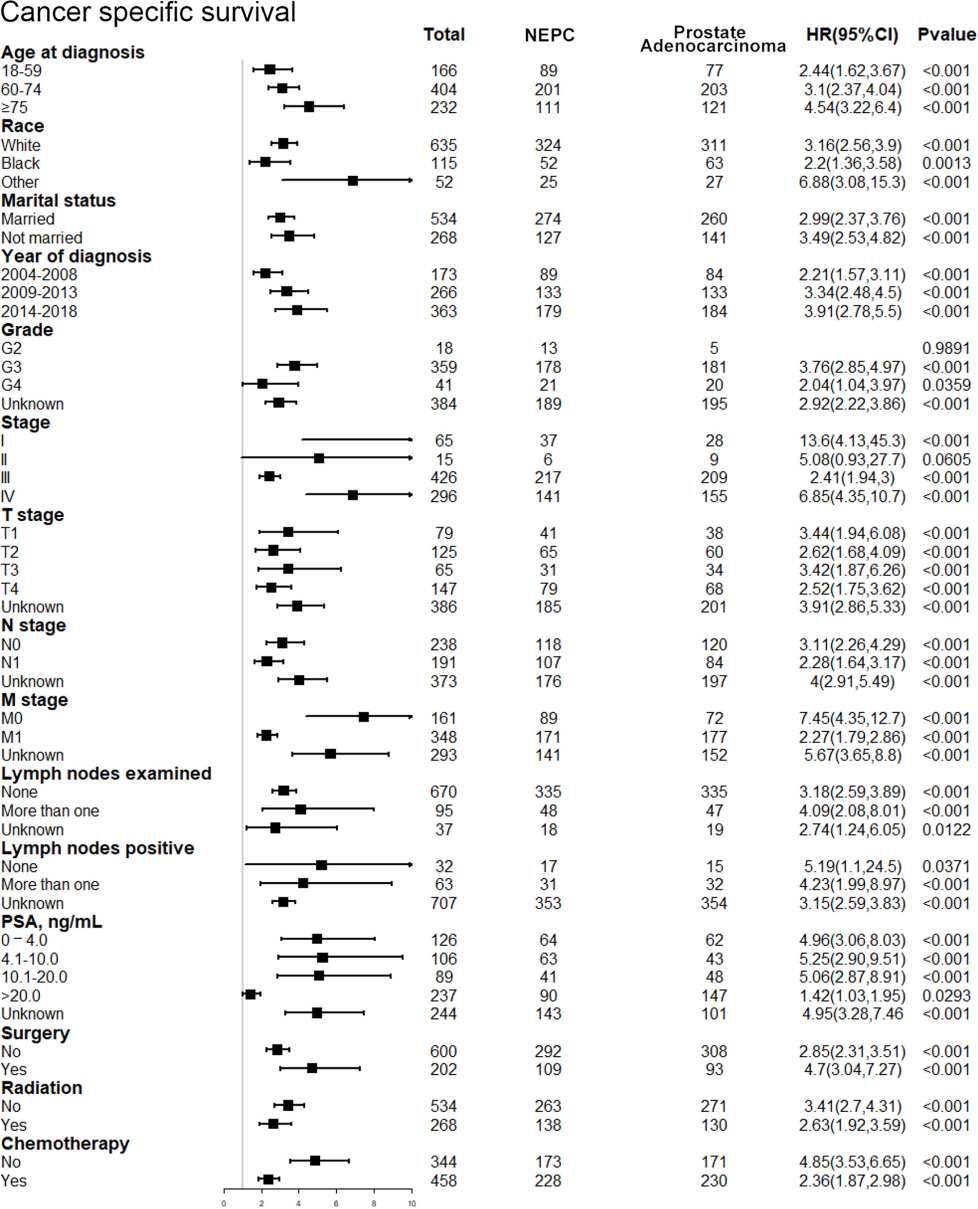
Forest plot of the subgroup analysis for NEPC and prostate adenocarcinoma in CSS. NEPC, neuroendocrine prostate cancer; CSS, cancer-specific survival.

After discovering the shortened survival of NEPC patients, we next aimed to evaluate the prognostic consistency and difference in diverse subgroups of prostate cancer patients between NEC and adenocarcinoma patients (**Figure 3**). According to the baseline demographic and clinicopathologic characteristics, NEC and adenocarcinoma patients were divided into subgroups, respectively. The results demonstrated that NEC patients obtained significantly poorer prognosis than adenocarcinoma patients across all subgroups except for G2 (HR = 3.25, 95%CI=0.68–15.4, P=0.1371), stage II (HR = 2.56, 95%CI=0.64–10.2, P=0.1831) and lymph nodes negative (HR = 3.57, 95%CI=0.94–13.4, P=0.0602) subgroups. We suspected that the insufficient sample size may contribute to no statistic difference of the three subgroups above. Nonetheless, the general tendency for the worse survival outcomes were existing in NEPC patients. Similar results were shown in subgroup analysis for CSS (**Figure 4**).

**Figure 5:**
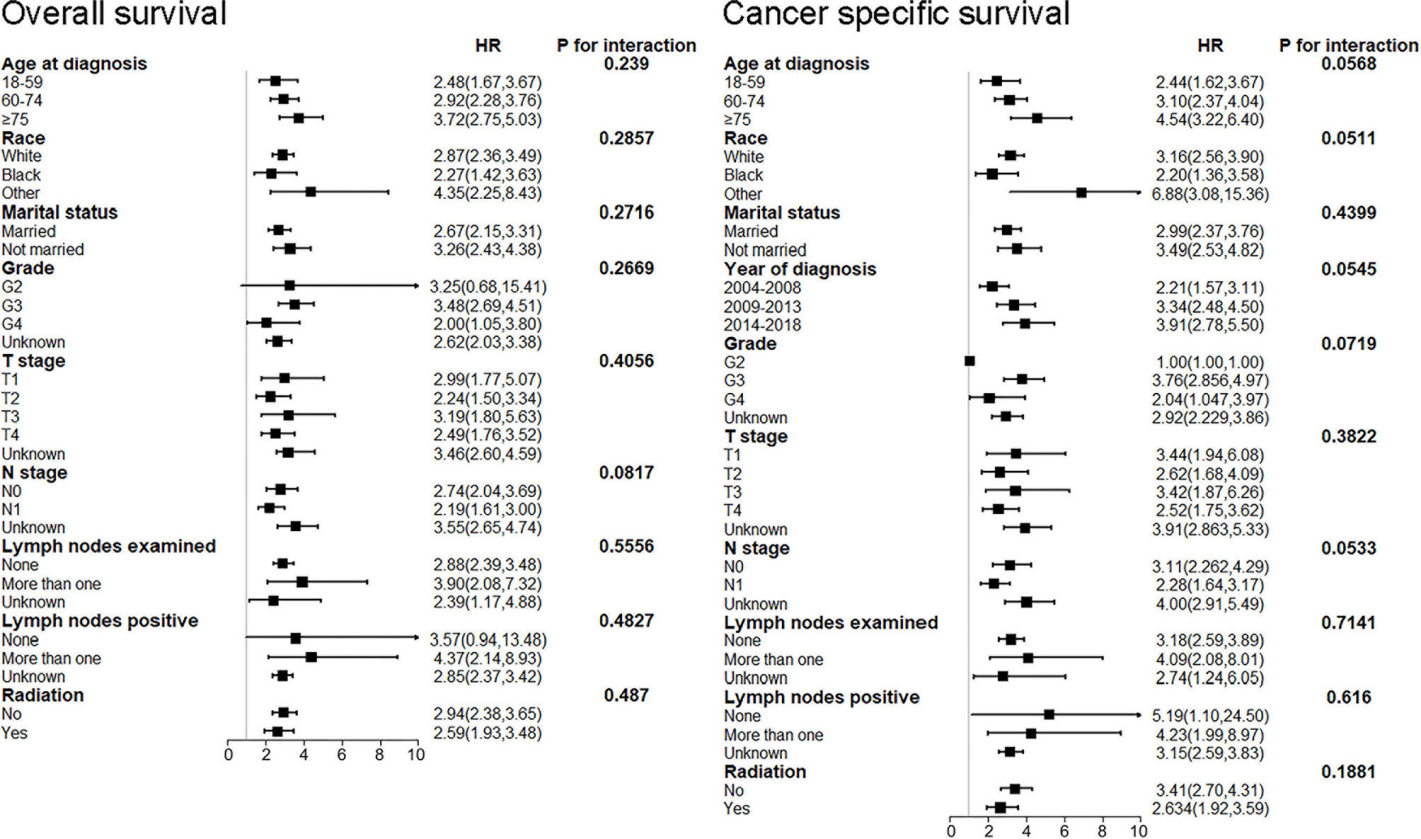
Subgroup analysis for interaction between NEPC and potential covariates in both OS and CSS. NEPC, neuroendocrine prostate cancer; OS, overall survival; CSS, cancer-specific survival.

Furthermore, we performed subgroup analysis to test the interaction after adjusting for the potential covariates (**Figure 5**). No significant difference was found for age at diagnosis, race, marital status, grade, T stage, N stage, lymph nodes examined, lymph nodes positive, radiation in both OS and CSS. The results uncovered that NEPC patients had a poorer survival outcome out of all subgroups. These results indicated that among patients with prostate cancer, the histological subtype of NEC had poorer prognosis than adenocarcinoma, which was not affected by other potential variates. Especially, it was reasonable to speculate that the histological subtype of NEC was an independent prognostic factor for patients with prostate cancer.

## Discussion

Our study is the most representative and comprehensive of the latest primary survival information of NEC compared with the most common histological type of prostate cancer. Given that NEPC is a rare and highly aggressive malignancy, majority of investigations are based on case reports or retrospective studies limited by small sample sizes (13–17). Consequently, the present study performed an investigation of a prostate cancer patient cohort based on large population from SEER registries between 2004 and 2018. We aimed to compare the survival outcomes of NEC with adenocarcinoma among prostate cancer patients according to clinicopathologic characteristics and explore the prognostic values in NEPC. Several meaningful conclusions could be obtained from our study. Among patients with prostate cancer, NEC had a worse prognosis than adenocarcinoma, even after adjustment for potential covariates. Moreover, subgroup analysis suggested that NEC patients obtained significantly poorer survival outcomes than adenocarcinoma patients across almost all subgroups. Last but not the least, there was no interaction among age at diagnosis, race, marital status, year of diagnosis, grade, T stage, N stage, lymph nodes examined, lymph nodes positive, radiation and the histological subtype of NEC was an independent prognostic factor for prostate cancer.

Although NEPC is a rare entity, the incidence rates of it maintained an upward trend in recent years (18). It had risen by approaching 6.8% per year, which could be mainly attributed to advanced medical technology and improved diagnostic methods (10, 18). Specially, the incident rates of small cell carcinoma (SCC) had a similar increasing trend of nearly 7.0% per year (18). Previous studies revealed that it was quite possible that the rise in incidence of NEPC was driven by SCC (19–21). On the other hand, several studies hold the view that the utilization of ADT was related to the incidence of NEPC (22–24). ADT was a primary therapy for prostate cancer targeting the androgen axis, which was first put forward by Huggins and Hodges in 1941 (11). Recently, the incidence rates of NEPC rose accompanied by the utilization of highly potent ADT, such as abiraterone and enzalutamide before or after chemotherapy for CRPC (25, 26). Long-term androgen deprivation could promote adenocarcinoma cells lose androgen receptor (AR) expression and eventually developed to NEC cells, which was called treatment-related NEPC (t-NEPC) (10, 27). However, it was reported that the utilization of ADT obviously decreased in 2004 and 2005 while the incident rates of NEPC, by contrast, displayed an increasing trend (19). Hence, such hypothesis is still not exactly elucidated. The upward incident trend of NEPC were supposed to be highlighted and the issue of long-term exposure to ADT in the clinic was warranted to be resolved in the coming years.

In the present study, NEC patients with PSA levels higher than 4.0 ng/mL accounted for 43.7%, compared with 72.9% of adenocarcinoma patients. This result suggested that except for loss of AR, NEPC patients are typically manifested by the downregulation of PSA (28). Our investigation was consistent with previous studies, which demonstrated that the PSA marker was usually expressed in adenocarcinoma while SCC, large cell carcinoma, or mixed adenocarcinoma neuroendocrine histology were scarcely expressed PSA (29). Hence, the low or non-rising serum PSA levels in tumor cells may indicate a relatively poorer prognosis (30). It also implied that serum PSA screening may not be effective for detection of NEPC in the clinic (7). The US Preventive Services Task Force (USPSTF) has recommended against PSA screening first in 2008 for men aged 75 years and older and then in 2012 for all men. However, since USPSTF’s 2012 recommendation, the incidence of advanced-stage prostate cancer has continued to rise though rates of localized disease have declined (6). Currently, the diagnosis of NEPC is mainly according to metastatic tumor biopsy confirming tumor morphology. Although there were no standard criteria for the best opportunity to conduct tumor biopsy, the NCCN guidelines recommended performing metastatic biopsy in suspected patients with particularly atypical spread, aggressive characteristics, and/or development with low serum PSA levels (30). Serum NE markers like CgA and NSE levels as well as synaptophysin (SYP), chromogranin and CD56 were classic biomarkers of NE cell, which were frequently upregulated in NEPC by immunohistochemistry (IHC), but neither of them was necessary for the diagnosis of NEPC in the clinic (31, 32). In order to achieve early diagnosis and effective treatment, it will be crucial to confirm feasible biomarkers that can detect the emergence of NEPC transformation during sequential therapies. A further investigation of biological characteristics of NEPC is indispensable to overcome the obstacle of this highly malignant prostate cancer.

The prevalent therapeutic modalities for prostate adenocarcinoma patients mainly include surgical removal of the prostate (radical prostatectomy), or radiation therapy with or without ADT. For early-stage or localized tumors, radical prostatectomy or radiation therapy is potentially effective and safe treatment option (33). ADTs is still first-line treatment for metastatic prostate cancer. However, after initial response to ADT, the tumor develops an androgen-insensitive form known as CRPC (34). ARPIs including abiraterone, enzalutamide, apalutamide and darolutamide have been developed for CRPC treatment. Nevertheless, partial ARPI- resistant CRPC may eventually develop NEPC due to AR- independent mechanisms in prostate cancer.

Our study suggested that the median OS of NEPC patients was only 12 months compared with 42 months of prostate adenocarcinoma patients. The severe invasiveness and the delayed diagnosis contributed to the final poor survival outcomes of NEPC. For example, we found that NEPC patients had an extremely high rate of metastasis, accounted for 45.4% of the group. In addition, the proportion of receiving surgery treatment for NEPC patients was significantly lower than prostate adenocarcinoma patients due to patients in advanced stage missing optimal opportunity for surgery. Until now, radical resection and palliative resection are the primary treatment for early NEPC without distal metastasis (35). Currently, the first- line treatment for NEPC is platinum- based chemotherapy, such as a combination of cisplatin and etoposide (36, 37). Cisplatin‐or etoposide‐based systemic chemotherapies, combined with surgery or radiation is the main therapy for NEPC with metastasis currently (38). The initial response of NEPC to chemotherapy is considerable. Unfortunately, its limitations are obvious: high and short response duration owing to acquired drug resistance (36). However, the effect of systemic treatment is not so satisfactory. Accurate assessment, early diagnosis and timely treatment of NEPC is critical for enhancing the clinical effect and thereby improving the prognosis.

Considering that the poor prognosis of NEPC is overwhelming, the novel effective therapeutic methods aiming at specific targets is warranted to be explored. Currently, emerging molecular targets with in the landscape of NEC differentiation put insight into individual therapy for NEPC. Rearrangement of TMPRSS2–ERG in NEPC was a crucial finding to prove that NEPC is evolved from conventional prostate adenocarcinoma (39). In the progression of evolution, several underlying molecular mechanism function, including loss of AR and tumor suppressors (TP53, PTEN, RB1) and induction of neural programs (39, 40). Especially, activation of mitotic programs such as Aurora kinase A (AURKA) upregulation and MYCN amplification are involved. AURKA, associated with MYCN amplification could regulates the assembly of mitotic spindle apparatus and eventually influences chromosome separation (41, 42). In addition, epigenetics regulation changes play an important role as well. Transcription factor RE1-silencing transcription factor (REST), suppressing neuronal differentiation, was found to be downregulated in 50% NEPC (43). Furthermore, microenvironment changes including endogenous IL-6 expression (44), MMP-9 production and other pro-inflammation cytokines upregulation fulfil complicated and comprehensive function in the process of adenocarcinoma transdifferentiating into NEC (45). Correspondingly, AURKA inhibitor PHA-739358 (danusertib) was confirmed to be effective on the growth of NE tumor cells and mouse xenograft models (46). This kinase inhibitor is being evaluated in phase II clinical trials and is expected to be applied for individual therapy prospectively in the clinic (46). Besides, other promising therapeutic targets for NEPC are also currently undergoing investigation in clinical trials, such as rocalpituzumab tesirine (DLL3 inhibitor) (47), GSK126 (EZH2 inhibitor) (48), and avelumab (immune-checkpoint PDL1 inhibitor) (49). Therefore, the remarkable progress in the molecular mechanism of NEPC established the foundation for the new effective treatment.

Peptide receptor radionuclide therapy (PRRT) is considered a curative and safe treatment option for NEPC (50). NEC cells have a higher expression of somatostatin receptors (SSTRs) than normal cells, which renders SSTR2 a potential target for NEPC treatment. The radiolabelled (Lutetium-177 or Yttrium-90) somatostatin analogues (SSAs) can target SSTR subtypes on the tumor cell surface and cause DNA damage in the cell nucleus which subsequently leads to cell death (51). Currently, 177Lu-DOTATATE or 177Lu-oxodotreotide is registered for the treatment of progressive and advanced grade 1–2 NEPC (50). On the other hand, 177Lu-PSMA-617 targets prostate-specific membrane antigen (PSMA), a cell-surface protein enriched in prostate cancer, which is used to treat metastatic prostate cancer (52). Besides, Radium-223 (223Ra) is another radiopharmaceutical treatment for patients with metastatic castration resistant prostate cancer patients (mCRPC) with symptomatic bone metastases and no known visceral metastatic disease (53). However, no research has showed that 223Ra could be performed in the treatment of NEPC.

*De novo* NEPC is a rare clinical entity, accounting for approximately 1% of all prostate cancers. Correspondingly, t-NEPC occurs in 10–17% of patients with CRPC by developing resistance to ADT and/or APRI treatment (54). The managements for the two types of NEPC are not identical and the difference in the details should attract enough attention (55). For locally advanced *de novo* NEPC, radiation therapy and radical resection are usually recommended. Given that majority of *de novo* NEPC patients present with distal metastatic disease at diagnosis, platinum-based chemotherapy should be adopted rather than ADT or APRI treatment (56). Previous researched suggested that t-NEPC occur in approximately 30% of metastatic CRPC, which suggests a strong possibility of distal metastasis at diagnosis. Thus, radiation therapy or radical resection is not recommended generally for t-NEPC. Considering prostate adenocarcinoma admixed with extensive neuroendocrine differentiation in t-NEPC, a trial of ADT in combination with cytotoxic chemotherapy is recommended. The chemotherapy regimens for *de novo* NEPC are usually platinum plus etoposide combinations. However, t-NEPC is frequently treated with docetaxel or a combination of carboplatin plus docetaxel rather than etoposide. Because docetaxel is an effective chemotherapeutic agent both for neuroendocrine and the adenocarcinoma components (56).

Due to the rarity of NEPC, our study conducted a retrospective study enrolling 482 patients with NEPC from the SEER. Thus, based on a large population, we had sufficient cases to make more credible and valuable analyses. Moreover, we provided the latest and comprehensive clinicopathological information of NEPC according to the recent released database. Nevertheless, our study had several limitations. Firstly, the detailed information such as chemotherapy regimens and operational styles were not available from the SEER, which was a severe obstacle for us to estimate the effect of treatment and assess the survival outcomes. Secondly, the retrospective nature of the study caused unavoidable selection biases, although PSM was performed. Thirdly, the ADT exposure history can’t be provided by the SEER. This factor is a critical variable for investigating the issue about adenocarcinoma transdifferentiates into NEC.

## Conclusion

The results of our study suggested that the prognosis of NEC was worse than that of adenocarcinoma among prostate cancer patients, even after adjustment for demographic and clinicopathological characteristics by PSM. Subgroup analysis further demonstrated that NEPC patients obtained significantly poorer prognosis than prostate adenocarcinoma patients across nearly all subgroups. Besides, the histological subtype of NEC was an independent prognostic factor for prostate cancer.

## Data Availability Statement

The dataset from SEER database generated and/or analyzed during the current study are available in the SEER dataset repository (https://seer.cancer.gov/).

## Ethics Statement

The data from SEER are publicly available and de-identified.

## Author Contributions

JPY: designed this research, completed the data analysis statistics work, and wrote the manuscript. YL: conception and design, collection and assembly of data, and data analysis and interpretation. XL, JS, YZ, and JY: worked in data collection and analysis. MZ: guided all research work, reviewed the manuscript, and provided financial support. All authors contributed to the article and approved the submitted version.

## Funding

This work was supported by grants from National Natural Science Foundation of China (U20A20348, 81871646) and Key R & D Projects of Zhejiang Province (2021C03039).

## Conflict of Interest

The authors declare that the research was conducted in the absence of any commercial or financial relationships that could be construed as a potential conflict of interest.

## Publisher’s Note

All claims expressed in this article are solely those of the authors and do not necessarily represent those of their affiliated organizations, or those of the publisher, the editors and the reviewers. Any product that may be evaluated in this article, or claim that may be made by its manufacturer, is not guaranteed or endorsed by the publisher.

## References

[1] Siegel RL Miller KD Fuchs HE Jemal A. Cancer Statistics, 2021. CA Cancer J Clin (2021) 71(1):7–33. doi: 10.3322/caac.21654 33433946

[2] Sung H Ferlay J Siegel RL Laversanne M Soerjomataram I Jemal A. Global Cancer Statistics 2020: GLOBOCAN Estimates of Incidence and Mortality Worldwide for 36 Cancers in 185 Countries. CA Cancer J Clin (2021) 71(3):209–49. doi: 10.3322/caac.21660 33538338

[3] Aggarwal R Huang J Alumkal JJ Zhang L Feng FY Thomas GV. Clinical and Genomic Characterization of Treatment-Emergent Small-Cell Neuroendocrine Prostate Cancer: A Multi-Institutional Prospective Study. J Clin Oncol (2018) 36(24):2492–503. doi: 10.1200/JCO.2017.77.6880 PMC6366813 29985747

[4] Aggarwal R Zhang T Small EJ Armstrong AJ. Neuroendocrine Prostate Cancer: Subtypes, Biology, and Clinical Outcomes. J Natl Compr Canc Netw (2014) 12(5):719–26. doi: 10.6004/jnccn.2014.0073 24812138

[5] Beltran H Tomlins S Aparicio A Arora V Rickman D Ayala G. Aggressive Variants of Castration-Resistant Prostate Cancer. Clin Cancer Res (2014) 20(11):2846–50. doi: 10.1158/1078-0432.CCR-13-3309 PMC4040316 24727321

[6] Fillon M. Rates of Advanced Prostate Cancer Continue to Increase. CA Cancer J Clin (2020) 70(6):427–9. doi: 10.3322/caac.21641 32986246

[7] Marcus DM Goodman M Jani AB Osunkoya AO Rossi PJ. A Comprehensive Review of Incidence and Survival in Patients With Rare Histological Variants of Prostate Cancer in the United States From 1973 to 2008. Prostate Cancer Prostatic Dis (2012) 15(3):283–8. doi: 10.1038/pcan.2012.4 22349984

[8] Terry S Beltran H. The Many Faces of Neuroendocrine Differentiation in Prostate Cancer Progression. Front Oncol (2014) 4:60. doi: 10.3389/fonc.2014.00060 24724054 PMC3971158

[9] Wang ZA Toivanen R Bergren SK Chambon P Shen MM. Luminal Cells are Favored as the Cell of Origin for Prostate Cancer. Cell Rep (2014) 8(5):1339–46. doi: 10.1016/j.celrep.2014.08.002 PMC4163115 25176651

[10] Wang HT Yao YH Li BG Tang Y Chang JW Zhang J. Neuroendocrine Prostate Cancer (NEPC) Progressing From Conventional Prostatic Adenocarcinoma: Factors Associated With Time to Development of NEPC and Survival From NEPC Diagnosis-a Systematic Review and Pooled Analysis. J Clin Oncol (2014) 32(30):3383–90. doi: 10.1200/JCO.2013.54.3553 25225419

[11] Huggins C Hodges CV. Studies on Prostatic Cancer: I. The Effect of Castration, of Estrogen and of Androgen Injection on Serum Phosphatases in Metastatic Carcinoma of the Prostate. 1941. J Urol (2002) 168(1):9–12. doi: 10.1016/S0022-5347(05)64820-3 12050481

[12] Beltran H Jendrisak A Landers M Mosquera JM Kossai M Louw J. The Initial Detection and Partial Characterization of Circulating Tumor Cells in Neuroendocrine Prostate Cancer. Clin Cancer Res (2016) 22(6):1510–9. doi: 10.1158/1078-0432.CCR-15-0137 PMC4990782 26671992

[13] Kumar K Ahmed R Chukwunonso C Tariq H Niazi M Makker J. Poorly Differentiated Small-Cell-Type Neuroendocrine Carcinoma of the Prostate: A Case Report and Literature Review. Case Rep Oncol (2018) 11(3):676–81. doi: 10.1159/000493255 PMC6243899 30483097

[14] Hu J He T Jin L Li Y Zhao Y Li W. Pure Small-Cell Carcinoma of the Prostate Presenting With Increasing Prostate-Specific Antigen Levels: A Case Report and Review of the Literature. Mol Clin Oncol (2018) 9(2):197–200. doi: 10.3892/mco.2018.1644 30101021 PMC6083420

[15] Weprin S Yonover P. Small Cell Carcinoma of the Prostate: A Case Report and Brief Review of the Literature. Urol Case Rep (2017) 13:61–2. doi: 10.1016/j.eucr.2016.10.010 PMC5408139 28462157

[16] Whitaker DA Jr Miller DH Jagadesh N Strong GW Hintenlang L Schenk WB. Small Cell Carcinoma of the Prostate in an Elderly Patient: A Case Report and Review of the Literature. Rare Tumors (2016) 8(4):6657. doi: 10.4081/rt.2016.6657 28191295 PMC5226053

[17] Alves D Calmeiro ME Silva R Coelho H. Small-Cell Neuroendocrine Cancer of the Prostate: An Atypical Presentation of a Common Disease. BMJ Case Rep (2016) 2016:bcr2016216199. doi: 10.1136/bcr-2016-216199 PMC5073671 27707760

[18] Alanee S Moore A Nutt M Holland B Dynda D El-Zawahry A. Contemporary Incidence and Mortality Rates of Neuroendocrine Prostate Cancer. Anticancer Res (2015) 35(7):4145–50. 26124369

[19] Elliott SP Jarosek SL Wilt TJ Virnig BA. Reduction in Physician Reimbursement and Use of Hormone Therapy in Prostate Cancer. J Natl Cancer Inst (2010) 102(24):1826–34. doi: 10.1093/jnci/djq417 PMC3001964 21131577

[20] Papandreou CN Daliani DD Thall PF Tu SM Wang X Reyes A. Results of a Phase II Study With Doxorubicin, Etoposide, and Cisplatin in Patients With Fully Characterized Small-Cell Carcinoma of the Prostate. J Clin Oncol (2002) 20(14):3072–80. doi: 10.1200/JCO.2002.12.065 12118020

[21] Schron DS Gipson T Mendelsohn G. The Histogenesis of Small Cell Carcinoma of the Prostate. An Immunohistochemical Study. Cancer (1984) 53(11):2478–80. doi: 10.1002/1097-0142(19840601)53:11<2478::AID-CNCR2820531119>3.0.CO;2-Q 6324985

[22] Potosky AL Haque R Cassidy-Bushrow AE Ulcickas Yood M Jiang M Tsai HT. Effectiveness of Primary Androgen-Deprivation Therapy for Clinically Localized Prostate Cancer. J Clin Oncol (2014) 32(13):1324–30. doi: 10.1200/JCO.2013.52.5782 PMC3992722 24638009

[23] Keating NL O’Malley AJ McNaughton-Collins M Oh WK Smith MR. Use of Androgen Deprivation Therapy for Metastatic Prostate Cancer in Older Men. BJU Int (2008) 101(9):1077–83. doi: 10.1111/j.1464-410X.2007.07405.x PMC2900629 18190632

[24] Terry S Maillé P Baaddi H Kheuang L Soyeux P Nicolaiew N. Cross Modulation Between the Androgen Receptor Axis and Protocadherin-PC in Mediating Neuroendocrine Transdifferentiation and Therapeutic Resistance of Prostate Cancer. Neoplasia (2013) 15(7):761–72. doi: 10.1593/neo.122070 PMC3689239 23814488

[25] Scher HI Fizazi K Saad F Taplin ME Sternberg CN Miller K. Increased Survival With Enzalutamide in Prostate Cancer After Chemotherapy. N Engl J Med (2012) 367(13):1187–97. doi: 10.1056/NEJMoa1207506 22894553

[26] Fizazi K Scher HI Molina A Logothetis CJ Chi KN Jones RJ. Abiraterone Acetate for Treatment of Metastatic Castration-Resistant Prostate Cancer: Final Overall Survival Analysis of the COU-AA-301 Randomised, Double-Blind, Placebo-Controlled Phase 3 Study. Lancet Oncol (2012) 13(10):983–92. doi: 10.1016/S1470-2045(12)70379-0 22995653

[27] Vlachostergios PJ Papandreou CN. Targeting Neuroendocrine Prostate Cancer: Molecular and Clinical Perspectives. Front Oncol (2015) 5:6. doi: 10.3389/fonc.2015.00006 25699233 PMC4313607

[28] Wang J Xu W Mierxiati A Huang Y Wei Y Lin G. Low-Serum Prostate-Specific Antigen Level Predicts Poor Outcomes in Patients With Primary Neuroendocrine Prostate Cancer. Prostate (2019) 79(13):1563–71. doi: 10.1002/pros.23878 31376193

[29] Simon RA di Sant’Agnese PA Huang LS Xu H Yao JL Yang Q. CD44 Expression is a Feature of Prostatic Small Cell Carcinoma and Distinguishes it From Its Mimickers. Hum Pathol (2009) 40(2):252–8. doi: 10.1016/j.humpath.2008.07.014 18835619

[30] Yamada Y Beltran H. Clinical and Biological Features of Neuroendocrine Prostate Cancer. Curr Oncol Rep (2021) 23(2):15. doi: 10.1007/s11912-020-01003-9 33433737 PMC7990389

[31] Epstein JI Amin MB Beltran H Lotan TL Mosquera JM Reuter VE. Proposed Morphologic Classification of Prostate Cancer With Neuroendocrine Differentiation. Am J Surg Pathol (2014) 38(6):756–67. doi: 10.1097/PAS.0000000000000208 PMC4112087 24705311

[32] Fléchon A Pouessel D Ferlay C Perol D Beuzeboc P Gravis G. Phase II Study of Carboplatin and Etoposide in Patients With Anaplastic Progressive Metastatic Castration-Resistant Prostate Cancer (mCRPC) With or Without Neuroendocrine Differentiation: Results of the French Genito-Urinary Tumor Group (GETUG) P01 Trial. Ann Oncol (2011) 22(11):2476–81. doi: 10.1093/annonc/mdr004 21436186

[33] Patel GK Chugh N Tripathi M. Neuroendocrine Differentiation of Prostate Cancer-An Intriguing Example of Tumor Evolution at Play. Cancers (Basel) (2019) 11(10):1405. doi: 10.3390/cancers11101405 PMC6826557 31547070

[34] Kaarijärvi R Kaljunen H Ketola K. Molecular and Functional Links Between Neurodevelopmental Processes and Treatment-Induced Neuroendocrine Plasticity in Prostate Cancer Progression. Cancers (Basel) (2021) 13(4):692. doi: 10.3390/cancers13040692 33572108 PMC7915380

[35] de Bono JS Logothetis CJ Molina A Fizazi K North S Chu L. Abiraterone and Increased Survival in Metastatic Prostate Cancer. N Engl J Med (2011) 364(21):1995–2005. doi: 10.1056/NEJMoa1014618 21612468 PMC3471149

[36] Wang Y Wang Y Ci X Choi SYC Crea F Lin D. Molecular Events in Neuroendocrine Prostate Cancer Development. Nat Rev Urol (2021) 18(10):581–96. doi: 10.1038/s41585-021-00490-0 PMC10802813 34290447

[37] Yamada Y Beltran H. Clinical and Biological Features of Neuroendocrine Prostate Cancer. Curr Oncol Rep (2021) 23(2):15. doi: 10.1007/s11912-020-01003-9 33433737 PMC7990389

[38] Beer TM Armstrong AJ Rathkopf DE Loriot Y Sternberg CN Higano CS. Enzalutamide in Metastatic Prostate Cancer Before Chemotherapy. N Engl J Med (2014) 371(5):424–33. doi: 10.1056/NEJMoa1405095 PMC4418931 24881730

[39] Logothetis CJ Gallick GE Maity SN Kim J Aparicio A Efstathiou E. Molecular Classification of Prostate Cancer Progression: Foundation for Marker-Driven Treatment of Prostate Cancer. Cancer Discovery (2013) 3(8):849–61. doi: 10.1158/2159-8290.CD-12-0460 PMC3926428 23811619

[40] Kani K Malihi PD Jiang Y Wang H Wang Y Ruderman DL. Anterior Gradient 2 (AGR2): Blood-Based Biomarker Elevated in Metastatic Prostate Cancer Associated With the Neuroendocrine Phenotype. Prostate (2013) 73(3):306–15. doi: 10.1002/pros.22569 22911164

[41] Mosquera JM Beltran H Park K MacDonald TY Robinson BD Tagawa ST. Concurrent AURKA and MYCN Gene Amplifications Are Harbingers of Lethal Treatment-Related Neuroendocrine Prostate Cancer. Neoplasia (2013) 15(1):1–10. doi: 10.1593/neo.121550 23358695 PMC3556934

[42] Otto T Horn S Brockmann M Eilers U Schüttrumpf L Popov N. Stabilization of N-Myc is a Critical Function of Aurora A in Human Neuroblastoma. Cancer Cell (2009) 15(1):67–78. doi: 10.1016/j.ccr.2008.12.005 19111882

[43] Lapuk AV Wu C Wyatt AW McPherson A McConeghy BJ Brahmbhatt S. From Sequence to Molecular Pathology, and a Mechanism Driving the Neuroendocrine Phenotype in Prostate Cancer. J Pathol (2012) 227(3):286–97. doi: 10.1002/path.4047 PMC3659819 22553170

[44] Lee SO Chun JY Nadiminty N Lou W Gao AC. Interleukin-6 Undergoes Transition From Growth Inhibitor Associated With Neuroendocrine Differentiation to Stimulator Accompanied by Androgen Receptor Activation During LNCaP Prostate Cancer Cell Progression. Prostate (2007) 67(7):764–73. doi: 10.1002/pros.20553 17373716

[45] Jin RJ Lho Y Connelly L Wang Y Yu X Saint Jean L. The Nuclear factor-kappaB Pathway Controls the Progression of Prostate Cancer to Androgen-Independent Growth. Cancer Res (2008) 68(16):6762–9. doi: 10.1158/0008-5472.CAN-08-0107 PMC2840631 18701501

[46] Meulenbeld HJ Bleuse JP Vinci EM Raymond E Vitali G Santoro A. Randomized Phase II Study of Danusertib in Patients With Metastatic Castration-Resistant Prostate Cancer After Docetaxel Failure. BJU Int (2013) 111(1):44–52. doi: 10.1111/j.1464-410X.2012.11404.x 22928785

[47] Puca L Gavyert K Sailer V Conteduca V Dardenne E Sigouros M. Delta-Like Protein 3 Expression and Therapeutic Targeting in Neuroendocrine Prostate Cancer. Sci Transl Med (2019) 11(484). doi: 10.1126/scitranslmed.aav0891 PMC6525633 30894499

[48] Dardenne E Beltran H Benelli M Gayvert K Berger A Puca L. N-Myc Induces an EZH2-Mediated Transcriptional Program Driving Neuroendocrine Prostate Cancer. Cancer Cell (2016) 30(4):563–77. doi: 10.1016/j.ccell.2016.09.005 PMC5540451 27728805

[49] Isaacsson Velho P Antonarakis ES. PD-1/PD-L1 Pathway Inhibitors in Advanced Prostate Cancer. Expert Rev Clin Pharmacol (2018) 11(5):475–86. doi: 10.1080/17512433.2018.1464388 PMC6317331 29641940

[50] Minczeles NS Hofland J de Herder WW Brabander T. Strategies Towards Improving Clinical Outcomes of Peptide Receptor Radionuclide Therapy. Curr Oncol Rep (2021) 23(4):46. doi: 10.1007/s11912-021-01037-7 33721105 PMC7960621

[51] La Salvia A Espinosa-Olarte P Riesco-Martinez MDC Anton-Pascual B Garcia-Carbonero R. Targeted Cancer Therapy: What’s New in the Field of Neuroendocrine Neoplasms? Cancers (Basel) (2021) 13(7):1701. doi: 10.3390/cancers13071701 33916707 PMC8038369

[52] Cuda TJ Riddell AD Liu C Whitehall VL Borowsky J Wyld DK. PET Imaging Quantifying (68)Ga-PSMA-11 Uptake in Metastatic Colorectal Cancer. J Nucl Med (2020) 61(11):1576–9. doi: 10.2967/jnumed.119.233312 32358088

[53] Sciuto R Rea S Ungania S Testa A Dini V Tabocchini MA. The Role of Dosimetry and Biological Effects in Metastatic Castration-Resistant Prostate Cancer (mCRPC) Patients Treated With (223)Ra: First in Human Study. J Exp Clin Cancer Res (2021) 40(1):281. doi: 10.1186/s13046-021-02056-9 34488829 PMC8420003

[54] Zaffuto E Pompe R Zanaty M Bondarenko HD Leyh-Bannurah SR Moschini M. Contemporary Incidence and Cancer Control Outcomes of Primary Neuroendocrine Prostate Cancer: A SEER Database Analysis. Clin Genitourin Cancer (2017) 15(5):e793–800. doi: 10.1016/j.clgc.2017.04.006 28506524

[55] Conteduca V Oromendia C Eng KW Bareja R Sigouros M Molina A. Clinical Features of Neuroendocrine Prostate Cancer. Eur J Cancer (2019) 121:7–18. doi: 10.1016/j.ejca.2019.08.011 31525487 PMC6803064

[56] Aggarwal R Zhang T Small EJ Armstrong AJ. Neuroendocrine Prostate Cancer: Subtypes, Biology, and Clinical Outcomes. J Natl Compr Canc Netw (2014) 12(5):719–26. doi: 10.6004/jnccn.2014.0073 24812138

